# Do technologies distract users? Investigating technology experience vs. user wellbeing

**DOI:** 10.3389/fpsyg.2026.1775004

**Published:** 2026-06-10

**Authors:** Abdul Waheed Siyal, Ren Lei

**Affiliations:** School of Digital Economics and Management, Wuxi University, Wuxi, China

**Keywords:** digital transformation, distractions, parallel communications, technology consumption experience, time pressure

## Abstract

Digital transformation, on one end, has made life more compatible in the ultra-fast-growing world but, on the other end, has caused many complexities in the form of technology interruption and distraction. During the technology consumption experience, individuals receive multiple communications from personal and professional counterparts, which trigger their instinct to respond in between the original task. Consequently, distractions divert users' focus and waste valuable time portion. Current research addresses the call to contribute to the literature about the disruptive roles of communication technologies underlining technology consumption experiences. We used SmartPLS to perform data analysis. For study 1, we examined the impact of parallel communications in putting people under time constraints, which incapacitated individuals from appropriate technology consumption experience. In study 2, we investigated the possible effects of parallel communication in distracting people from the original task and deteriorating the technology consumption experience. Results revealed that parallel communications triggered distractions and time pressure. In either situational behavior, individuals experience technology differently, which might occur due to time constraints or being distracted from the original task. In both cases, it becomes difficult for people to get back to the original task with the same flow, pace, or cognitive understanding, which deteriorates the technology consumption experience. The findings provide insights for theory and practice. In general, the research on adverse effects of communication technologies is scarce and specifically rare on behavioral aspects of parallel communications impacting technology consumption experience. Current research addresses the call to contribute to the literature on the disruptive roles of communication technologies in the context of technology consumption experiences.

## 1 Introduction

The digital transformation which has necessitated uninterrupted online connectivity has deeply influenced individuals and their experiences of technology consumption (Akdim et al., 2022; Alzaidi and Agag, 2022; Buck et al., 2023; Truţa et al., 2023). This constant connectivity has generated several adverse effects on individuals' technology consumption experiences due to parallel communications between their original tasks (Kim et al., 2022; Leroy and Glomb, 2018; Sharma et al., 2023). To some extent, the multitasking ability could somehow balance several tasks being performed at a time, but multitasking itself creates mental fatigue, which ultimately deteriorates the technology consumption experience (Kudesia et al., 2022). The prevalence and affordability of communication technologies have led to constant connectivity being an acceptable norm in the online community in all walks of life (Tarafdar et al., 2019). Hence, multiple communications could probably distract individuals from the original goal by impairing task flow, interest, and enjoyment (Dust et al., 2022; Fu et al., 2020; Rusou et al., 2020).

Technologies are expected to boost performance and productivity by enabling people to process and share information (Mishra et al., 2022; Naeem, 2021), but at the same time, they distract people by diverting their attention off the original task and generating time constraints (Orhan et al., 2021). For example, when individuals face multiple communications from managers, colleagues, friends, and family members or any other social network, such interruptions, and intrusions trigger their fear of missing out (FOMO) (Farivar et al., 2022; Rosen and Samuel, 2015), and they feel a sort of compelled to check in between their ongoing experience (Li et al., 2023). Such counterproductive behavior causes distraction, which takes approximately 23 min to fully recover and resume the main goal (Zhang et al., 2023). Earlier research has covered interruptions in the context of workplaces where employees are allowed to bring their own devices therefore, easily fall prey to distractions which negatively impact their relative work performance by slowing down progress on original tasks (Duan et al., 2023; Swider et al., 2022; Zou and Ren, 2022).

Prior research has mainly focused on technostress (Fleming et al., 2022; Kim et al., 2022), technophobia (Califf and Brooks, 2020), technoaddiction (Fu et al., 2020), internal and external connectivity using advanced information technology (Ye et al., 2023), technology overload in terms of job stress, low performance and low job satisfaction (Brown et al., 2022; Buck et al., 2023; Nastjuk et al., 2023; Xu et al., 2022), information usefulness (Chen et al., 2023), constant connectivity (Reinke and Gerlach, 2022), negative impacts of communication technology (Sharma et al., 2023), technology use and digital divides (Mehra et al., 2020), resilience and digital transformation (Mishra et al., 2022), Impromptu communication technology vs. work overload (Stich et al., 2018), job performance (Orhan et al., 2021), digital platform functional dimensions (Ramasundaram et al., 2023), interruption timings and their relative effects on performance (Kus et al., 2022). Studies exploring technology consumption experience affected by parallel communication and time convenience are very scarce (Chekembayeva et al., 2023; Chiang, 2022; Klaus, 2022). When consumers are experiencing a technology, such disruptions could provoke them to respond in between their ongoing consumption activity and would deteriorate their consumption experience as such interruptions cause repeated distractions (Yin et al., 2018). The amount of time wasted during these disruptions distracts the consumption flow, thereby putting time pressure on the users, which ruins the overall consumption experience. The two-study design enables a more holistic exploration of the impact of parallel communication on the technology consumption experience via different but complementary mechanisms. Study 1 tests the role of time pressure as a situational constraint due to simultaneous communications. Study 2 extends the model by investigating distractions as an alternative cognitive mechanism for the same relationship. The study investigates these mechanisms in two different studies, which strengthens the robustness and explanatory power of the proposed framework and provides a more comprehensive understanding of the different ways in which parallel communications can diminish technology consumption experience.

Current research addresses the call to contribute to the literature on the disruptive roles of communication technologies in the context of technology consumption experiences. The objective of Study 1 is to examine the impact of parallel communications in putting people under time constraints that incapacitate individuals from appropriate technology consumption experience. This study also explores possible indirect effects of time pressure between parallel communications and technology consumption experience. Study 2 investigates the possible effects of parallel communication in distracting people from the original task and deteriorating technology consumption experience therein. This study also explores possible indirect effects of distractions between parallel communications and technology consumption experience. Both studies examine hypothetical behaviors triggered by parallel communications defined as time pressure and or distractions from the ongoing task. In either situational behavior, individuals experience technology differently, which might occur due to time constraints, or being distracted away from the original task. In both situations, it becomes difficult for people to get back to the original task with the same flow, pace, or cognitive understanding, which deteriorates the technology consumption experience.

The remainder of this article is structured as follows. Section 2 reviews the literature and hypotheses. Section 3 addresses the design, procedure, results, and discussions for study 1. Section 4 addresses the design, procedure, results, and discussions for study 2. Section 5 presents general discussions, implications, conclusions, limitations, and prospects.

## 2 Theoretical foundation and development of the hypothesis

This study is grounded in the Stimulus–Organism–Response (S–O–R) framework (Mehrabian and Russell, 1974) and cognitive load theory (Sweller, 1988; Sweller et al., 2011). The integration of these two theoretical perspectives provides a comprehensive explanation of how digitally mediated communication environments influence users' internal cognitive states and, subsequently, their technology consumption experience. The S–O–R framework posits that environmental stimuli (S) affect individuals' internal states (O), which in turn shape their behavioral or experiential responses (R) (Mehrabian and Russell, 1974). In this study, parallel communications are conceptualized as the primary stimulus, representing a communication environment in which individuals engage with multiple, simultaneous information streams. Such environments are characteristic of contemporary digital platforms and are likely to impose significant cognitive demands on users. Within the organism component, this study focuses on two cognitive states: distractions and time pressure. These states reflect the internal processing conditions triggered by exposure to parallel communications. Distractions capture the fragmentation of attention caused by competing information inputs, while time pressure reflects the perceived urgency to process and respond to multiple communication demands within a limited time.

To explain these organism-level effects more precisely, cognitive load theory is incorporated. Cognitive load theory suggests that individuals have limited working memory capacity, and when the cognitive demands of a task exceed this capacity, performance and experience deteriorate (Sweller, 1988; Sweller et al., 2011). In the context of this study, parallel communications increase extraneous cognitive load by introducing multiple, simultaneous information cues that compete for attentional resources. This overload leads to higher levels of distraction and perceived time pressure, as individuals struggle to allocate limited cognitive resources effectively across tasks. The response component of the model is technology consumption experience, which reflects users' overall evaluation of their interaction with technology. According to both S–O–R and cognitive load theory, excessive cognitive load and attentional fragmentation negatively influence experiential outcomes, reducing the quality, efficiency, and satisfaction associated with technology use. By integrating the S–O–R framework with cognitive load theory, this study provides a theoretically grounded explanation of how external communication environments translate into internal cognitive strain and ultimately shape user experience. While S–O–R offers the overarching structure linking stimuli, internal states, and responses, cognitive load theory specifies the underlying cognitive mechanisms through which these effects occur. This combined perspective strengthens the model's theoretical rigor and provides a clear justification for the proposed relationships.

### 2.1 Parallel communications and technology consumption experience

Parallel communication refers to individuals' use of multiple gadgets at a time or multiple tasks over one device simultaneously. Multiple information processing poses challenges in efficiently handling different information data at a time (Sharma et al., 2023; Zhang et al., 2022). Since every user has a different cognitive capability, parallel communications at a time would overload information and create confusion, leading to erroneous decisions (Mohammed et al., 2022). Parallel communications expose individuals to simultaneous stimuli, fragmenting attention and increasing cognitive interference, consistent with the stimulus–organism mechanism of S–O–R. Recent studies show that multitasking environments significantly heighten attentional disruption due to increased extraneous cognitive load (Xu et al., 2026; Zhou et al., 2025). In the context of communication technology use, users face impromptu interactions that not only discontinue their ongoing technology consumption but also overload information and cause disruption (Yin et al., 2018). The interruption causes severe cognitive disruptions, and since the interruption was not initiated by the subject user, such disruptions cause delays in responding to earlier technology in use (Choi et al., 2023; Heitmayer, 2022) thereby affecting consumption flow and experience. Moreover, it also compels users to respond to the interruption in their ongoing consumption flow (Orhan et al., 2021).

Parallel communication environments create expectations of rapid responses across channels, intensifying perceived time pressure as an internal cognitive state. From a cognitive load perspective, handling concurrent inputs compresses perceived time availability and increases urgency (Maskun et al., 2025; Matthews et al., 2025). Being disconnected from the digital world is no longer a viable option because these days, either professional or personal matters are being carried out through communication technologies with portable devices, which facilitate endless inflow that can interrupt and distract users anywhere, thereby provoking digital overload (Labban and Bizzi, 2022; Talwar et al., 2020). According to the conclusions of (Labban and Bizzi 2022), the technology consumption experience merely depends on how users perceive the applicability of the technology in question; therefore, technology itself is not something problematic but its usage patterns might be. For example, users incapacitated by FOMO (fear of missing out) (Pang, 2021) would, in between their ongoing technology consumption experience, respond to the interruptions or intrusions and easily let themselves be distracted (Leroy et al., 2020).

Prior research has mainly focused on technology overload in terms of job stress, low performance and low job satisfaction (Duan et al., 2023; Nastjuk et al., 2023; Xu et al., 2022), constant connectivity (Reinke and Gerlach, 2022), internal and external connectivity using advanced information technology (Ye et al., 2023), information usefulness (Chen et al., 2023), cognitive change in online health communities (Gu et al., 2023), technostress (Kim et al., 2022), technophobia (Califf and Brooks, 2020), technoaddiction (Fu et al., 2020), technology use and digital divides (Mehra et al., 2020), distraction effect on political and entertainment content (Matthes et al., 2023), smartphone zombie behavior (Pressey et al., 2023), digital platform functional dimensions (Ramasundaram et al., 2023), resilience and digital transformation (Mishra et al., 2022), negative impacts of communication technology (Sharma et al., 2023), Impromptu communication technology vs. work overload (Stich et al., 2018), job performance (Orhan et al., 2021), interruption timings and their relative effects on performance (Kus et al., 2022). Studies exploring technology consumption experience affected by parallel communication and time convenience are very scarce (Chekembayeva et al., 2023). Studies exploring technology consumption experiences affected by parallel communication are very scarce. When consumers are experiencing a technology, such disruptions could provoke them to respond in between their ongoing consumption activity and would deteriorate their consumption experience as such interruptions cause repeated distractions (Yin et al., 2018). Recently (Shafqat et al., 2023) argued that individuals redirect their time and efforts toward experiences within social circles. More recently, (Wang et al., 2023) established that technology pitfalls and social constructivism depend on the mechanism individuals adopt to use it. Similarly, (Deng and Jiang, 2023) portrayed social media influencers as antecedents of anxiety among users. According to the findings of (Maduku et al. 2023), an increased amount of anxiety weakens users' commitment to the original goal.

The amount of time wasted during these disruptions distracts the consumption flow, thereby putting time pressure on the users, which ruins the overall consumption experience. This is congruent with the findings of (Marsh et al. 2022) that interruptions waste time and energy and ultimately have negative effects on productivity. Based on the above rationale, we assume that parallel communication substantially distracts users and generates time pressure, which affects their technology consumption experience.

H1: Parallel communication generates time pressure, which ultimately affects the technology consumption experience.

H1a: Parallel communication distracts users from ongoing activity, which ultimately affects the technology consumption experience.

H1b: Parallel communication directly affects the technology consumption experience.

### 2.2 Distractions and time pressure

Distractions reduce attentional focus and cognitive processing efficiency, leading to poorer technology consumption experience within the organism–response pathway. Recent evidence suggests that higher cognitive interference significantly lowers user satisfaction and engagement in digital contexts (Chen et al., 2026; Halpin, 2026; USMAN and Abdulrahman, 2026). Distractions are referred to as irrelevant stimuli which divert attention away from the primary task. There can be several factors triggering distraction, such as emails, subscription alerts, tweets, and other social network group texts, causing users' control failure over the ongoing task or goal (Draheim et al., 2022). Alerts do allow users to choose whether to engage with it or not. For example, the receivers can overlook the communication, which could portray senders an impression of not being interested at the moment (Fellesson and Salomonson, 2020). But most people would respond within seconds' time. This may be because they want to present themselves as more responsible (Chang and Tang, 2015). According to the findings of (Leroy et al. 2020), 70 percent of people respond to the received emails within 6 s; they further argued that people tend to risk their lives to respond even when they are driving. This is also in congruence with (Montag and Walla, 2016) that frequent checking behaviors allow distractions and prevent ongoing technology consumption experience. Undoubtedly, social media in 21^st^ century has emerged as a primary source of information (Akdim et al., 2022; Arora et al., 2019; Ji et al., 2023) where users generate a variety of information from diverse sources in real-time context (Alzaidi and Agag, 2022) and simultaneously their interactions and perceptions also do exist in real-time on social media platforms (Zhuang et al., 2023). But despite several advantages, it has overloaded individuals with the amount of information that is beyond their capacity (Sharma et al., 2023), and such a high information overload might tend users toward information avoidance (Li, 2023; Sultana et al., 2023). This alarming situation disrupts users by creating time constraints, resulting in fear of missing out (FOMO), anxiety, and depression (Bermes, 2021; Li et al., 2023).

Perceived time pressure induces cognitive strain and hurried processing, negatively influencing experiential quality and decision satisfaction. Under high cognitive load conditions, users report diminished experience quality and increased stress during technology use (Tian et al., 2026; Yanamandra et al., 2026). Interruptions and intrusions very often use up valuable time without bringing any outputs, and this wastage causes time shortage and pressure (Leroy and Glomb, 2018); hence, users need to beware themselves to attend to the right information at the right time (Islam et al., 2021). For example, when people get distracted from primary tasks they consume their valuable time on less important or irrelevant tasks. And now, when they want to resume their ongoing task, they need to regain the focus from where they left off and overcome the resumption lag by remembering the steps completed and to be completed with an appropriate set of rules (Leroy et al., 2020). Here comes the time limitation which builds pressure and impairs original task synthesis and sense of progress that ultimately affects the technology experience (Ali et al., 2022). Such a techno-stress of processing overloaded information in a time-constraint situation impairs users' psychology and behavior, resulting in various emotional sufferings (Ketron et al., 2016) such as incapacitated consciousness and negative behavioral responses (Laato et al., 2020). According to the conclusions of (Leroy and Glomb 2018), when people assume that they have to resume the interrupted task under time pressure, the effects, such as task synthesis and sense of progress, become worse, which impairs the experience.

H2: Time pressure affects technology consumption experience.

H2a: Time pressure mediates between parallel communication and technology consumption experience.

H3: Distractions affect technology consumption experience.

H3a: Distractions mediate between parallel communication and technology consumption experience.

### 2.3 Overview of studies

We conducted two field surveys to evidence our predictions. Study1 examines the impact of parallel communications in putting people under time constraints, which incapacitate individuals from appropriate technology consumption experience. This study also explores possible indirect effects of time pressure between parallel communications and technology consumption experience. Study 2 investigates the possible effects of parallel communication in distracting people from the original task and deteriorating technology consumption experience therein. This study also explores possible indirect effects of distractions between parallel communications and technology consumption experience. Both studies examine hypothetical behaviors triggered by parallel communications defined as time pressure and or distractions from the ongoing task. In either situational behavior, individuals experience technology differently, which might occur due to time constraints, or being distracted away from the original task. In both situations, it becomes difficult for people to get back to the original task with the same flow, pace, or cognitive understanding, which deteriorates the technology consumption experience.

**Figure 1:**
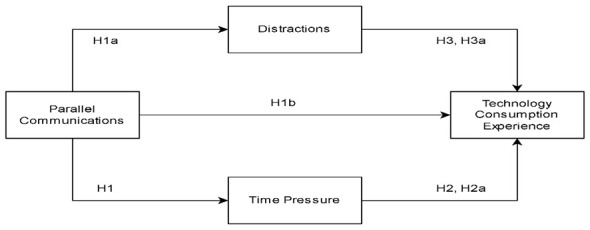
The theoretical research framework.

Although both studies examine the influence of parallel communications on the technology consumption experience, they are different in terms of their theoretical approach and the mechanisms they propose to explain these effects. While in Study 1, the focus is on time pressure as the key mechanism through which parallel communications have a negative impact on the technology consumption experience, Study 2 looks at the role of distractions as a different cognitive mechanism underlying the same relationship. By isolating these two mechanisms in distinct studies, the research provides a more robust and complete picture of how parallel communications degrade people's engagement, continuity, and overall experience of technology consumption activities. Study 1 shows that parallel communications negatively affect the technology consumption experience through the mechanism of time pressure. Study 2 explores distractions as a cognitive mechanism that can lead to the same effect. The research, by separating these two mechanisms into individual studies, offers a clearer and more complete understanding of how parallel communications negatively impact people's engagement, continuity, and overall experience of technology consumption activities. Figure 1 presents the theoretical research framework (For further details on item-scales refer to Appendix A).

### 2.4 Study 1

The objective of study 1 is to test the relationship between parallel communication and time pressure (H1), Parallel communications and technology consumption experience (H1b), Time pressure and technology consumption experience (H2), and the indirect effect of time pressure between parallel communication and technology consumption experience (H2a).

### 2.5 Participants and procedure

The study aimed to highlight the role of parallel communication in triggering time pressure that could substantially affect consumers' technology experience. To meet our purpose, we recruited 150 professionals (65 female and 85 male) who were aged above 22 years and belonged to diverse professional fields. The minimum academic qualification of participants was a graduate degree and above. For further demographic details, refer to Table 1. The recruited participants were explained the research purpose and motivated to participate as a social responsibility. These participants were also frequent users of online social platforms in Pakistan, which could further add value to determining the impact of time pressure triggered by parallel communications, including both professional and personal communications, which ultimately affect the technology consumption experience.

**Table 1:** Respondent demographic characteristics.

Demographic variables	Frequency	Percentage
Gender		
Male	80	57
Female	60	43
Age		
22–25	23	15
26–30	34	24
31–35	34	24
36–40	27	19
41–45	13	09
46–50	09	06
50+	05	03
Education		
Graduate	42	35
Masters	98	65
Platform		
Facebook; WhatsApp and Google+	127	91
Instagram	13	09
Platform experience		
3 years−4 years	17	12
5 years−6 years	45	32
More than 6 years	78	56

The survey consisted of two parts. In part one, participants were interviewed about their demographic information and past-experience on technology consumption. In the second part, participants were interviewed about item scales adapted from past studies. We adopted two items for parallel communication from (Orhan et al., 2021), three items for time pressure (Ryari et al., 2021), and four items for technology consumption experience from (Leroy et al., 2020; Orhan et al., 2021). We slightly modified the adopted items scales to fit the research purpose for assessing the role of multiple communications at a time and their time pressure impact on technology consumption experience. The item scales were measured with seven-point Likert scales. It took 5 weeks to complete 207 samples for the study 1. 67 samples were deleted from the final dataset due to incomplete and/or inconsistent responses. Hence, we proceeded with 140 samples for further statistical analysis.

### 2.6 Analysis and results

We chose a two-step approach to carry out statistical analysis (Nikbin et al., 2015; Yusof et al., 2016). We began with model assessment and measurement with an evaluation of predictive relevance by testing proposed hypotheses. For validating convergent reliability, we confirmed inter-item reliability by assessing the factor loading of each item scale and average variance extracted (AVE). We also determined Cronbach's Alpha and rho to further strengthen the appropriateness of item scales internal consistency. Further, to refrain from multicollinearity issues, we confirmed variance inflated factors (VIF). The VIF results are in accordance with the guidelines of (Hair et al. 2011), which eliminates the prevalence of any possible multicollinearity issues in the current study. Table 2 presents the main statistics achieved for study 1.

**Table 2:** Main statistics.

Factor	Item	Factor loading	AVE	rhoA	Alpha	VIF	*R* square	*f* square
Parallel communication	PC1 PC2	0.869 0.894	0.777	0.719	0.714	2.083	–	0.303
Time pressure	TP1 TP2 TP3	0.871 0.856 0.884	0.758	0.842	0.840	1.000	0.520	1.083
technology consumption experience	TCE1 TCE2 TCE3 TCE4	0.858 0.893 0.910 0.878	0.783	0.912	0.907	2.083	0.723	0.428

We followed the guidelines of (Kock 2015) to confirm common method variance, which suggested that the VIF values below 3.3 do not indicate any CMV issues in the study. The maximum VIF value for study 1 was reported as 2.083, which is considerably below the suggested criteria and hence eliminates the possibility of CMV. To further determine CMV, we also ran Harman's single-factor test (Podsakoff and Organ, 1986). The stats reported the largest factor with 4.25%, with a cumulative variance of 19.08%. As a result, no single factor in both predictor and criterion factors reported any major variance (Podsakoff et al., 2012). Thereby rejecting any possibility of CMV in the current study, which is also in accordance with the guidelines of (Kumar 2012).

This study employs Partial Least Squares Structural Equation Modeling (PLS-SEM) using SmartPLS. Unlike covariance-based SEM, PLS-SEM is a variance-based approach that does not require multivariate normality (Hair et al., 2021). Nevertheless, to ensure data quality and robustness of results, preliminary data screening was conducted. Skewness and kurtosis values were examined and found to be within acceptable thresholds (± 2), indicating no severe deviations from normality. Additionally, the data were assessed for outliers and missing values, and no critical issues were identified. These checks support the suitability of the dataset for PLS-SEM analysis.

Furthermore, we investigated discriminant validity by assessing the square roots of AVE with correlation coefficients. All the predictor and criterion factors indicated considerable strength toward their respective correlation coefficients (Barclay et al., 1995; Fornell and Larcker, 1981). More detailed stats are presented in Table 3.

**Table 3:** Discriminant validity.

Factor	PC	TCE	TP
Parallel communication	0.882		
Technology consumption experience	0.777	0.885	
Time pressure	0.721	0.799	0.871

**Figure 2:**
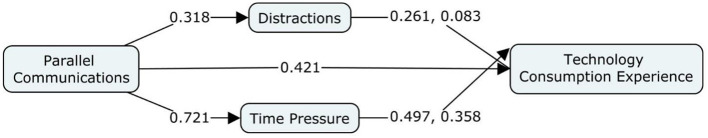
The structural results for study 1 and study 2.

We ran a bootstrapping procedure along with 5,000 samples in structural equating modeling to confirm path coefficients (Hair et al., 2011). Achieved stats empirically supported all the hypothesized relations indicated in Figure 2 and Table 4. The prediction ability of the framework was examined by calculating R2 with the PLS algorithm. The obtained R2 values for time pressure 0.520 and technology consumption experience 0.723 substantially surpassed the acceptable threshold of 0.010 in social sciences research (Falk and Miller, 1992). Finally, we ran a blindfolding procedure to cross-validate redundancy and confirm the *Q*2 relevance of endogenous constructs (Fornell, 1994). We achieved stats of 0.385 for time pressure and 0.558 for technology consumption experience. The achieved stats are considerably greater than zero; hence, our model successfully indicates its predictive relevance to the study, which is in accordance with the guidelines of (Chin 1998).

**Table 4:** Path coefficients and significances.

Hyp	Relationship	Beta	STDEV	*t*-value	*P* values	Confidence intervals		Decision
						2.5%	97.5%	
H1	PC-> TP	0.721	0.037	19.263	0.000	0.641	0.790	Supported
H1b	PC-> TCE	0.419	0.081	5.179	0.000	0.251	0.562	Supported
H2	TP-> TCE	0.497	0.085	5.871	0.000	0.345	0.661	Supported
H2a	PC-> TP- TCE	0.358	0.066	5.418	0.000	0.245	0.490	Supported

## 3 Discussions

Study 1 provides evidence to support H1, H1b, H2, and H2a, explaining that a high volume of communications at a point in time when the user is already engaged with an ongoing task generates unusual time pressure, which eventually affects the original task. Distracting factors like text/voice messages from social networks cause control failure over the original goal (Sumuer and Kaşikci, 2022) and incur time shortage (Leroy and Glomb, 2018). In addition, such interruptions also affect the quality of communications by ticking the clock. For example, in such cases, consumers may mistake any information regarding financial transactions over a system, resulting in a monetary loss, or miss any important information on either side which, besides deteriorating technology experience might affect their personal and professional lives. Even when there are no time constraints, multiple communications at a time are likely to jeopardize the consumption experience with technologies by affecting task synthesis, flow, and cognitive understanding of the original task (Yin et al., 2018). Research by (Leroy et al. 2020) has witnessed that 70% of people reply to online communications within 6 s, and at times, they don't even fear endangering their lives while replying to online communications. More recently, (Wang et al., 2023) established that technology pitfalls and social constructivism depend on the mechanism individuals adopt to use it. The findings discussed here also get theoretical support from earlier research studies by (Akdim et al. 2022); (Alzaidi and Agag 2022); (Arora et al. 2019); (Bermes 2021); (Draheim et al. 2022); (Fellesson and Salomonson 2020); (Gu et al. 2023); (Ji et al. 2023); (Leroy et al. 2020); (Orhan et al. 2021); (Sharma et al. 2023); (Stich et al. 2018).

### 3.1 Study 2

The objective of study 2 is to test the relationship between parallel communication and distractions (H1a), Parallel communications and technology consumption experience (H1b), Distractions and technology consumption experience (H3), and the indirect effect of distractions between parallel communication and technology consumption experience (H3a).

### 3.2 Participants and procedure

The study aimed to highlight the role of parallel communication in triggering distractions that could substantially affect consumers' technology experience. To meet our purpose, we recruited 130 participants (59 female and 71 male) who were aged above 22 years and belonged to diverse professional fields. The minimum academic qualification of participants was a graduate degree and above. For further demographic details, refer to Table 5. After due consent, the recruited participants were explained the research purpose and motivated to participate as a social responsibility. These participants were also frequent users of online social platforms in Pakistan, which could further add value to determine the impact of distractions triggered by parallel communications, including both professional and personal communications, which ultimately affect the technology consumption experience.

**Table 5:** Respondent demographic characteristics.

Demographic variables	Frequency	Percentage
Gender		
Male	71	57
Female	54	43
Age		
22–25	19	15
26–30	21	17
31–35	30	24
36–40	22	18
41–45	15	12
46–50	11	09
50 +	07	05
Education		
Graduate	42	34
Masters	83	66
Platform		
Facebook; WhatsApp & Google+	107	86
Instagram	18	14
Platform experience		
3 years−4 years	20	16
5 years−6 years	39	31
More than 6 years	66	53

The field survey participation consisted of two parts. In part one, we interviewed participants about their demographic information and past experience with technology consumption. In part two, we interviewed participants about items scales adapted from past studies. We adopted two items for parallel communication from (Orhan et al., 2021), three items for distractions (Abbas et al., 2014; Orhan et al., 2021), and four items for technology consumption experience from (Leroy et al., 2020; Orhan et al., 2021). We slightly modified the adopted items‘ scales to fit the research purpose for assessing the role of multiple communications at a time and their distracting impact on the technology consumption experience. The items' scales were measured with seven-point Likert scales. It took 5 weeks to complete 207 samples. 82 samples were deleted from the final dataset due to incomplete and inconsistent responses. Hence, we proceeded with a 125 sample size for further statistical analysis.

## 4 Analysis and results

We chose a two-step approach to carry out statistical analysis (Nikbin et al., 2015; Yusof et al., 2016). We began with model assessment and measurement with an evaluation of predictive relevance by testing proposed hypotheses. For validating convergent reliability, we confirmed inter-item reliability by assessing the factor loading of each item scale and average variance extracted (AVE). We also determined Cronbach's Alpha and rho to further strengthen the appropriateness of item scales internal consistency. Further, to refrain from multicollinearity issues, we confirmed variance inflated factors (VIF). The VIF results are in accordance with the guidelines of (Hair et al. 2011), which eliminates the prevalence of any possible multicollinearity issues in the current study. Table 6 presents the main statistics achieved for study 2. We followed the guidelines of (Kock 2015) to confirm common method variance, who suggested that the VIF values below 3.3 do not indicate any CMV issues in the study. The maximum VIF value for study 2 was reported as 1.113, which is considerably below the suggested criteria hence eliminating the possibility of CMV. To further determine CMV, we also ran Harman's single-factor test (Podsakoff and Organ, 1986). The stats reported the largest factor with 4.11%, with a cumulative variance of 18.15%. As a result, no single factor in both predictor and criterion factors reported any major variance (Podsakoff et al., 2012). Thereby rejecting any possibility of CMV in the current study, which is also in accordance with the guidelines of (Kumar 2012).

**Table 6:** Main statistics.

Factor	Item	Factor loading	AVE	rhoA	Alpha	VIF	*R* square	*f* square
Parallel communication	PC1 PC2	0.934 0.859	0.885	0.883	0.765	1.000	–	0.113
Distractions	DI1 DI2 DI3	0.927 0.914 0.902	0.836	0.904	0.902	1.113	0.101	0.079
Technology Consumption Experience	TCE1 TCE2 TCE3 TCE4	0.921 0.876 0.888 0.885	0.796	0.929	0.915	1.113	0.224	0.118

This study employs Partial Least Squares Structural Equation Modeling (PLS-SEM) using SmartPLS. Unlike covariance-based SEM, PLS-SEM is a variance-based approach that does not require multivariate normality (Hair et al., 2021).

Nevertheless, to ensure data quality and robustness of results, preliminary data screening was conducted. Skewness and kurtosis values were examined and found to be within acceptable thresholds (± 2), indicating no severe deviations from normality. Additionally, the data were assessed for outliers and missing values, and no critical issues were identified. These checks support the suitability of the dataset for PLS-SEM analysis.

Furthermore, we investigated discriminant validity by assessing the square roots of AVE with correlation coefficients. All the predictor and criterion factors indicated considerable strength toward their respective correlation coefficients (Barclay et al., 1995; Fornell and Larcker, 1981). More detailed stats are presented in Table 7.

**Table 7:** Discriminant validity.

Factor	DI	PC	TCE
Distractions	0.914		
Parallel communication	0.318	0.897	
Technology consumption experience	0.318	0.403	0.892

We ran a bootstrapping procedure along with 5,000 samples in structural equating modeling to confirm path coefficients (Hair et al., 2011). Achieved stats empirically supported all the hypothesized relations indicated in Figure 2 and Table 8. The prediction ability of the framework was examined by calculating R2 with the PLS algorithm. The obtained R2 values for distractions 0.101 and technology consumption experience 0.224 substantially surpassed the acceptable threshold of 0.010 in social sciences research (Falk and Miller, 1992). Finally, we ran a blindfolding procedure to cross-validate redundancy and confirm the *Q*2 relevance of endogenous constructs (Fornell, 1994). We achieved stats of 0.080 for distractions and 0.166 for technology consumption experience. The achieved stats are considerably greater than zero; hence, our model successfully indicates its predictive relevance to the study, which is in accordance with the guidelines of (Chin 1998).

**Table 8:** Path coefficients and significances.

Hyp	Relationship	Beta	STDEV	*t*-value	*P* values	Confidence intervals		Decision
						2.5%	97.5%	
H1a	PC-> DI	0.318	0.084	3.777	0.000	0.149	0.486	Supported
H1b	PC-> TCE	0.320	0.087	3.684	0.000	0.150	0.480	Supported
H3	DI-> TCE	0.261	0.091	2.854	0.004	0.081	0.436	Supported
H3a	PC-> DI- TCE	0.083	0.039	2.149	0.032	0.019	0.174	Supported

## 5 Discussions

Study 2 provides evidence to support H1a, H1b, H3, and H3a by explaining that a high volume of communications at a point in time when the user is already engaged with an ongoing task distracts from the original task. Distracting factors like calls, emails, texts, and voice messages from social networks cause control failure over the original goal (Robinson and Steyvers, 2022). Multiple interruptions also affect the quality of communications. For example, the disruptions divert attention away from the original goal and cause resumption lag in regaining focus on the original task. Users need to recall the steps completed and or remaining to be completed (Leroy et al., 2020). This impairs original task synthesis and a sense of progress that ultimately affects the technology experience (Dust et al., 2022). This overburdening information, which is beyond users' processing capacity, might tend users toward information avoidance (Li, 2023; Sultana et al., 2023). This finding is in line with the conclusions of (Bermes, 2021; Ketron et al., 2016; Leroy and Glomb, 2018). When people assume that they have to resume the interrupted task, the effects, such as a lower sense of progress, flow, and interest, worsen the experience. In such cases, there are higher chances of misreporting or mistaking the information exchange, which might cause any financial or reputational loss on either side which, besides deteriorating technology experience, might affect their personal and professional life. Using multiple communication channels is more likely to jeopardize task flow and cognitive understanding of the original goal (Yin et al., 2018). This is also in congruence with (Montag and Walla, 2016) that frequent checking behaviors allow distractions and prevent ongoing technology consumption experience.

In line with prior research on technostress, our findings can be further understood through the distinction between technostress creators and inhibitors. Consistent with (Castillo et al., 2023) technology-related stressors such as overload, invasion, and constant connectivity deplete individuals' cognitive and psychological resources, thereby impairing task performance and experience. In our context, parallel communications function similarly to technostress creators by intensifying time pressure and distraction, ultimately reducing users' capacity to maintain focus and flow during technology consumption. Moreover, the emphasis by (Castillo et al. 2023) on technostress inhibitors, such as organizational support, training, and user involvement, highlights that the negative effects observed in this study are not inevitable. Instead, they can be mitigated through structured communication norms, improved technological support systems, and user-centered digital practices. This alignment not only strengthens the theoretical grounding of our findings but also underscores the importance of balancing technological demands with supportive mechanisms to enhance user experience.

Prevailing research specifically addresses interruption timings and their relative effects on performance (Kus et al., 2022; Orhan et al., 2021), constant connectivity (Reinke and Gerlach, 2022), technology overload in terms of job stress, low performance and low job satisfaction (Nastjuk et al., 2023; Xu et al., 2022), Impromptu communication technology vs. work overload (Stich et al., 2018), negative impacts of communication technology (Sharma et al., 2023), The research studies focusing on disruptive roles of communication technologies and technology consumption experience remain very scarce (Chekembayeva et al., 2023). This research aims to add value to users‘ perceptions because multiple communications between ongoing tasks cause repeated distractions (Nisafani et al., 2020; Yin et al., 2018). According to the findings of (Maduku et al. 2023), an increased amount of anxiety weakens users' commitment to the original goal. Similarly, (Deng and Jiang, 2023) portrayed social media influencers as antecedents of anxiety among users. Recently (Shafqat et al., 2023) argued that individuals redirect their time and efforts toward experiences within social circles.

Hence, anchoring on the adverse effects, this research answers a call to address the disruptive roles of communication technologies (Leroy and Glomb, 2018; Leroy et al., 2020). The research formulates an overarching framework to examine the adverse effects of parallel communications in terms of distractions and time constraints, which ultimately impair the technology consumption experience. Study 1 articulates that a high volume of communications in between an ongoing task generates unusual time pressure by ticking the clock. Further disrupting factors such as messages, updates, and alerts from social media weaken users' cognitive control over the ongoing activity, which adds to the shortage of time (Leroy and Glomb, 2018; Sumuer and Kaşikci, 2022). The paucity of time may tend users to rush and make erroneous decisions, which might result in financial and/or reputational loss. The results of Study 1 extend the findings of (Yin et al. 2018) that irrespective of time constraints, parallel communications still jeopardize synthesis, flow, and cognitive understanding of technology in consumption. Study 2 articulates that multiple communications divert attention away from the original goal and cause resumption lag in regaining focus on earlier tasks. Disruptions from social or professional networks cause control failure and affect task synthesis and quality of communication, which eventually affect the technology consumption experience (Dust et al., 2022; Robinson and Steyvers, 2022). Results of study 2 further explain that frequent checking behaviors allow distractions, which might tend users to mistake or misreport any information exchange. Such disruptions could jeopardize the technology experience and might affect users‘ personal and professional lives (Montag et al., 2021).

## 6 Implications

Theoretically, this research contributes to the literature by exploring the negative effects of parallel communications directly on technology consumption experience. This explains that when users keep on shifting their focus from the original task by responding to the communications initiated by other people while they are experiencing a technology. The deteriorating effects of parallel communications have also been theoretically enriched by studying its indirect effects through distracting users in the form of interruptions and intrusions or by putting users under time pressure through multiple communications at a time. Recent research has witnessed that distractions on one end impair original task achievement and, on the other end, put users under time constraints (Leroy and Glomb, 2018). Constant connectivity on professional and social networks has become inevitable in today's world. Despite several merits, it has caused immense time constraints and distractions to people. For example, the communicator could check the online availability of the receiver and determine the time taken to respond. This obligation has tended individuals to immediate responses to the digital communications received in between the ongoing tasks (Feldman and Greenway, 2021), which has proved to be detrimental to their performances with the ong^*^oing tasks (Puranik et al., 2020).

Our studies identify that multiple communications, either personal or professional, in between the ongoing tasks distract individuals from the original goal and put them under time constraints while resuming the original task, thereby affecting task flow and its cognitive understanding. This research separates the blurred boundaries for users to a better technology consumption experience by prioritizing the ongoing tasks rather than falling back on multitasking tendencies, which has failed to determine performance efficiency (Orhan et al., 2021). The tendency to be multitasking by immediately responding to communications in between the original tasks may cause higher costs to individuals by not only impairing the original goal but also with superficial outputs on the multitasked responses. Hence, users need to comprehend this phenomenon that simultaneous multiple connections consume more time in the shape of distractions and create time limitations that impair experiences by generating technology overload and counterproductive behaviors (Serenko and Bontis, 2016). Individuals need to evaluate their technology consumption experiences with and without multiple communications to see the differences in their personal and professional technology consumption experiences and adapt the respective behaviors by themselves rather than being regulated by other people like their managers, etc.

Learning to prioritize tasks would refrain users from mental health issues like addiction and anxiety and also could save individuals from digital diseases like FOMO (fear of missing out) and FOBO (fear of being offline) (Rosen and Samuel, 2015; Sharma et al., 2023). Therefore, it is important to aware individuals and society at large about the addictive technology use behavior that creates anxiety among users and compels them to switch their attention from important to unimportant or irrelevant communications, which ultimately impairs their experience with the technology.

Building on these insights, this study offers clear practical value for both individuals and organizations seeking to optimize technology use. For practitioners, the findings suggest the need to design structured communication norms such as designated response windows, prioritization protocols, and reduced expectations of immediacy to minimize unnecessary interruptions and cognitive overload. Organizations can translate these insights into policy by limiting non-essential real-time communications during deep-work periods and by promoting asynchronous collaboration tools that reduce time pressure. At the individual level, users can improve their technology consumption experience by consciously managing attention, disabling non-critical notifications, and adopting task segmentation strategies to preserve cognitive flow. Furthermore, digital platform designers may incorporate features that support focus management. For example, companies could adopt “do not disturb” modes or intelligent notification filtering. Collectively, these applications demonstrate that managing parallel communications is not merely a behavioral concern but a strategic lever to enhance productivity, wellbeing, and the overall quality of technology-enabled experiences.

## 7 Conclusion, limitations, and prospects

Current research aimed to investigate the impact of multiple communications from professional and social networks under the umbrella of parallel communications on the technology consumption experience. The study 1(*N* = 140) and study 2 (*N* = 125) opted to highlight the indirect effects of distractions and time pressure caused by multiple communications on the technology consumption experience. Study 1 provides a mechanism to refrain from the deteriorating role of multiple communications in the form of time wasters, which consume a large portion of important time and trigger time pressures and constraints on individuals that impair task flow and efficiency (Leroy and Glomb, 2018). Study 2 sheds light on how multiple communications in between the original task damage task synthesis and cognitive understanding and provides a mechanism to refrain from technology distractions and optimize technology experience (Serenko and Bontis, 2016).

Despite theoretical and practical contributions, present studies also suffer from several limitations which open new venues for researchers and scholars. Firstly, the field surveys for Studies 1 and 2 were carried out on the basis of self-reported measures gathered from the recruited participants in the field, which might be subject to any possible biases regarding participants' online connectivity and frequency with social and professional networks. Further, looking at the contextual nature of interruptions and distractions caused by communication technologies, their relevant effects may vary from case to case basis. Future studies could consider breaks and surprises (Leroy et al., 2020) as they may bring different outcomes against communications leading to distractions and time constraints. Further, moderating roles such as multi-tasking ability (Zhang and Rau, 2016), coping with mental fatigue from multitasking (Kudesia et al., 2022), and mindful approaches (Arslan and Coşkun, 2022) differentiating between males and females may bring different results on gender perceptions between multiple communications and technology consumption experience. In addition to physical and virtual disruptions (Nees and Fortna, 2015), interruptions could also be categorized into professional and personal to see their relevant impact on the technology consumption experience.

## Data Availability

Data will be available on demand via email request to the corresponding author at abdulwaheedsiyal@gmail.com.

## Appendix A

**Table A1:**

Item scales	
Parallel communications (Orhan et al., 2021)	On a typical workday, I engage in several online conversations at the same time.
	On a typical workday, I communicate with multiple people outside my work at the same time.
Time Pressure (Ryari et al., 2021)	In parallel communication, I am often under pressure.
	In parallel communication, I pay a lot of attention to time.
	In parallel communication, I feel under time pressure as the conversation continues longer.
Distractions (Abbas et al., 2014; Orhan et al., 2021)	While working, I have phone conversations.
	While working, I manually interact with my phone, like sending texts.
	While working, I check non-work-related emails on my personal email accounts.
Technology consumption experience from (Leroy et al., 2020; Orhan et al., 2021)	Sometimes the number of messages I receive makes me unproductive.
	I have many conflicting tasks (including non-work-related) at work that make it hard for me to focus on any one of them.
	I am immersed in my work.
	I get easily distracted from my plans at work.
